# Exercise, Osteoporosis, and Bone Geometry

**DOI:** 10.3390/sports5020029

**Published:** 2017-05-12

**Authors:** Amy T. Harding, Belinda R. Beck

**Affiliations:** 1 Menzies Health Institute Queensland, Griffith University, Gold Coast campus, Gold Coast 4222, Australia; amy.harding@griffithuni.edu.au; 2 School of Allied Health Sciences, Griffith University, Gold Coast campus, Gold Coast 4222, Australia; 3 The Bone Clinic, Coorparoo, Brisbane 4151, Australia

**Keywords:** bone geometry, exercise, older adults, osteoporosis, physical activity, review

## Abstract

Exercise is commonly recommended in the prevention and management of osteoporosis. The most common method to monitor bone mass and its response to interventions is bone densitometry. While closely associated with risk of fracture, densitometry-derived areal bone mineral density (aBMD) does not provide a reliable indication of bone geometry or morphological adaptation to stimuli. In fact, the effects of exercise interventions on aBMD are frequently modest, and may not fully represent the benefit of exercise to bone. Animal models suggest that mechanical loading indeed influences bone geometry and thus strength. Such an effect in humans has the potential to reduce osteoporotic fracture. The aim of the current narrative review is to provide an overview of what is known about the effects of exercise on bone geometry, with a focus on relevance to osteoporosis.

## 1. General Introduction

Osteoporosis is a progressive metabolic bone condition characterised by profound loss of skeletal mass, coupled with architectural deterioration, that increases bone fragility and susceptibility to fractures [1]. The epidemiological literature suggests close to 200 million individuals are afflicted by osteoporosis worldwide [2], with a degree of heterogeneity in prevalence by geographical region. Based on an overall prevalence of 10.3%, it was estimated that 10.2 million Americans 50 years and older have osteoporosis diagnosed from bone densitometry [3]. A further 43.4 million Americans over the age of 50 have low bone mass, or osteopenia [3]. Corresponding figures of osteoporosis prevalence in men and women over the age of 50 are 1.2 million Australians [4], 0.5 million Swiss [5], and 27.5 million in the European Union (cumulative total of the 27 member countries) [6]. Low trauma fractures, the main clinical consequence of the condition, occur at the hip, spine, wrist, and forearm, predominantly as a result of falling from standing height or less [7]. It has been suggested that osteoporosis causes 8.9 million fractures globally each year, at a rate of 1000 fractures per hour [8]. With the prevalence of low bone mass on the rise, population growth, and the significant increase in life expectancy, a commensurate increase in the incidence of low trauma fracture is to be expected. Based on projected demographic changes, globally the number of men and women over the age of 50 whom exceed the ‘threshold’ for major osteoporotic fracture is expected to increase from 157 million in 2010 to 319 million in 2040 [9]. Of all affected sites, hip fracture is regarded as the most debilitating, due to high morbidity and mortality [8], with associated pain, hospitalisation, surgery, loss of independence, and premature death [10,11]. For men over the age of 50, the remaining lifetime risk of osteoporotic fracture ranges from 13.1% to 22.4% [12,13], and the risk for women is almost twice as high, ranging from 39.7% to 51.4% [12,13,14,15].

Substantial experimental data from animal models has informed our understanding of the dynamic nature of bone mass and morphology, and the unique ability of bone to adapt to its mechanical environment. Within the context of osteogenic mechanical loading, the most critical characteristics are the magnitude [16,17], rate [18], and frequency [19,20,21] of engendered strain. Additional determinants of the osteogenic response are the direction and number of load applications [22], and dynamic rather than static loading is required [23]. To provide the maximal anabolic stimulus to bone, high magnitude strains should be applied at a rapid rate, and be of a varied nature. Only a small absolute number of load cycles need be applied to elicit an osteogenic response, and short bouts interspersed with recovery are preferable to long duration loading. With such a load protocol, the response of bone to habitual mechanical loading can thus theoretically be harnessed through targeted exercise to optimise bone mass during youth and ameliorate loss in old age. That is, the ideal osteogenic exercise program would include a variety of high load and high impact activities, applied with progressive overload.

Systematic reviews and meta-analyses have examined the effects of various exercise interventions on femoral neck, trochanter, total hip, and/or lumbar spine areal bone mineral density (aBMD; g/cm^2^) in middle aged and older men [24,25,26], and postmenopausal women [27,28,29,30,31,32]. Results of those analyses indeed suggest that a combination of high intensity weight-bearing exercise, particularly impact-inducing activities (hopping, bounding, and jumping) and progressive resistance training have a favourable, albeit modest, effect on bone mass at loaded sites. While findings support the notion that exercise should be recommended for individuals with osteoporosis, the optimal exercise program is yet to be identified. The ‘Too Fit To Fracture’ exercise recommendations for individuals with low bone mass or osteoporotic vertebral fracture specify progressive resistance training for each major muscle group at a target intensity of eight to twelve repetitions (i.e., moderate intensity), high challenge balance exercises to prevent falls, and moderate to vigorous aerobic exercise for general health benefits [33]. The recent Exercise and Sports Science Australia (ESSA) position statement on exercise prescription for the prevention and management of osteoporosis recommended a combined program of impact loading exercises, high to very high intensity progressive resistance training, and challenging balance training to reduce the risk of falls and fracture [34]. The intensity of each exercise modality that can be tolerated will vary depending on bone status (i.e., normal, low, or very low aBMD), the presence of clinical risk factors for falls, previous fracture/s, and contraindications to exercise [34].

In the past, exercise intervention studies for bone health have typically reported a primary outcome of dual-energy x-ray absorptiometry (DXA)-derived aBMD as an index of bone mass and strength. While true density is the ratio of mass to volume, aBMD is in fact a two-dimensional parameter derived from a three-dimensional structure as the apparent amount of bone tissue within the projected bone area. Although it is well accepted that low aBMD is associated with an increased risk of low trauma fracture (to the extent that for every one standard deviation reduction in aBMD the incidence of fracture increases two-fold [35], such that individuals with osteoporosis have the highest risk of incident fracture), the majority of low trauma fractures occur in individuals with osteopenia. In a population-based random sample of postmenopausal women over the age of 60 who underwent aBMD investigations a median of 5.6 years apart, 37.6%, 48.0%, and 14.5% exhibited normal, osteopenic, and osteoporotic total hip aBMD (based on World Health Organization criteria), respectively [36]. At study completion, 73.1% of all low trauma fractures occurred in women without osteoporosis (56.5% osteopenic and 16.6% with normal aBMD). It was also reported that women with osteopenia who sustained a fracture during the study time course had a lower probability of remaining fracture free over a five-year period than women with osteoporosis and no prevalent fracture. These findings highlight the inability of aBMD alone to account for whole bone strength and risk of fracture. Although risk of falling is a primary determinant of fracture risk, the contribution of bone morphology to bone strength should not be overlooked. The inability of aBMD to fully represent the structural elements of bone strength such as volumetric bone mineral density (vBMD; g/cm^3^), and cortical and trabecular bone architecture, is a major limitation of bone densitometry for the purposes of monitoring the response of bone to an exercise stimulus. Due to the non-linear relationship between bone area and volume, bone size can additionally affect estimations of density [37], and DXA can systematically underestimate bone mass in small bones [38]. DXA-derived aBMD accounts for the length and breadth of bone, but not its depth, providing an integrated areal measure, which may be confounded by inter-individual difference in bone size. In comparison, true vBMD determined using three-dimensional imaging techniques, captures bone length, breadth, and depth, such that the confounding effect of bone size is largely eliminated. Further, three-dimensional densitometric scanners yield discrete estimates of cortical and trabecular bone, and parameters of skeletal geometry (such as cortical width) that characterize bone strength to a greater extent than DXA.

Alongside age-related reductions in bone mass, deleterious changes to the spatial distribution (size and shape) of bone also become apparent; although long bones may partly compensate for loss of mass by increasing overall bone diameter via periosteal apposition [39]. Such changes in bone geometry can also occur in response to an osteogenic stimulus (i.e., exercise) independent of changes in aBMD, and will translate to marked increases in bone strength. The evolution of three-dimensional imaging technologies such as peripheral quantitative computed tomography (pQCT), quantitative computed tomography (QCT), high-resolution peripheral quantitative computed tomography (HR-pQCT), and Magnetic Resonance Imaging (MRI) has aided the quantification of morphological adaptation in response to exercise exposure. While the sensitivity of those instruments to detect subtle exercise-induced changes in bone geometry has not been exhaustively tested, the move to examine morphometric adaptation to loading is unquestionably a significant advance from the two dimensionality of DXA. Ultimately, monitoring changes in bone geometry beyond DXA-derived aBMD will provide further insight into the optimal therapeutic exercise program to enhance bone strength and reduce susceptibility to osteoporotic fracture.

## 2. Methods

A literature search was conducted identifying exercise intervention studies that reported bone geometric parameters determined using three-dimensional imaging technologies at any skeletal site. No participant age or sex restrictions were imposed during the search to encompass bone health across the lifespan. Reference lists of selected papers were hand searched to locate further studies of relevance to the review. Dissertations and abstracts were not included, nor were those trials that included disease-specific populations. The current review was restricted to bone geometry determined using three-dimensional imaging (pQCT, QCT, or MRI). Papers reporting areal bone strength indices or bone quality (e.g., aBMD, bone mineral content, and broadband ultrasound attenuation) from two-dimensional imaging technologies (DXA or quantitative ultrasonometry) in addition to three-dimensional techniques were included. Studies reporting only bone geometric parameters extracted from standard DXA scans were not included.

## 3. The Role of Physical Activity and Exercise on Bone Geometry across the Lifespan: Cross-Sectional Observations

There is strong evidence that physical activity plays an important role in accrual of bone mass during the growing years, including on bone structure. Although the precise optimal timing of exercise during childhood has yet to be determined, it is likely that the early pubertal years provide a particularly responsive window of opportunity. A recent review nevertheless concluded that exercise at all stages of skeletal development is likely to be beneficial [40]. Bone geometry is typically described in terms of volume, cross-sectional area (CSA), periosteal and endosteal diameters, and cortical and trabecular thickness. A number of paediatric and adult cross-sectional studies have reported bone geometry outcomes, as follows.

Within-subject, side-to-side comparison of bone parameters in sports with predominantly unilateral loading presents an opportune model to examine mechanoadaptation of bone. The study of young male and female racquet sport athletes confirms loading-related differences in the geometric properties of the humerus between playing and nonplaying arms [41]. Prepubertal male tennis players (11.3 ± 0.3 years), with an average 4.4-year training history, displayed significantly higher MRI-derived cortical and periosteal CSA at both the mid- and distal humerus in playing versus non-playing arms (+8.3% to +21.5%, *p* < 0.001) [41]. Even more pronounced side-to-side differences were observed in peripubertal boys (13.9 ± 0.2 years), with an average 6.8-year training history: cortical CSA was 20.1% and 32.7% higher at the dominant mid- and distal humeri, respectively. Cortical CSA was 18.3% and 22.5% higher at the same skeletal sites in postpubertal boys (17.1 ± 0.4 years) with approximately ten years of training. When pubertal stages were combined, training history was significantly positively associated with total bone CSA at both sites of the humerus (*r* = 0.37–0.43, *p* < 0.05). Others have observed similar cortical and periosteal bone CSA asymmetry due to the effect of loading on the playing arm in comparison to the nonplaying arm in pre-, peri-, and postpubertal girls [42].

Collegiate level jumping athletes (high or long jump) exhibited geometric differences between the jump and lead leg in tibial diaphyseal cortical CSA (+3.7%, *p* < 0.05) and thickness (+3.5%, *p* < 0.05), but not total CSA (+1.5%, *p* > 0.05) [43]. By contrast, there were no differences in bone geometry of the dominant and non-dominant leg of age-matched cross-country runners who load the lower extremity without a notable level of asymmetry. Thus, repetitive unilateral jumping-related forces on the lower extremity appear to preferentially thicken the cortex of the mid-shaft of the tibia.

Studies of younger athletes have observed similar differences in bone geometry, particularly in sports such as gymnastics which involve high impact loading of both the upper and lower extremities (vertical ground reaction forces of 3.5 (squat jump) to 10 times body weight (handstand with vertical push-off) [44]. Exposure to a minimum of five hours of artistic gymnastics training per week for at least two years between the age of eight and menarche (16.7 ± 2.1 years) was associated with elevated total and cortical bone CSA at the radial diaphysis and metaphysis, and metaphyseal trabecular bone CSA, in comparison to non-gymnasts (16.2 ± 2.2 years) [45]. Advantageous bone geometry has also been reported in the tibia of male sprint athletes with a long-term sprint training background, compared with age-matched non-athletic men [46]. Younger male sprint athletes (19 to 39 years) had significantly higher tibial mid-shaft total CSA than their age matched peers (588 ± 53 mm^2^ vs. 521 ± 65 mm^2^, *p* < 0.05), and a similar difference was present for older athletes (65 to 84 years) (562 ± 59 mm^2^ vs. 508 ± 48 mm^2^ *p* < 0.05). Mid-shaft cortical tibial CSA was higher in both younger (465 ± 47 mm^2^ vs. 399 ± 46 mm^2^, *p* < 0.05) and older (423 ± 41 mm^2^ vs. 383 ± 32 mm^2^, *p* < 0.05) sprint athletes than non-athletic referents. A comparison of national level female volleyball, hurdles, racquet sports, soccer, and swimming athletes with non-athletic referents revealed thicker distal tibial cortical walls in athletes than the non-athletes (+26.6% to +44.8%), with the exception of swimmers (−1.9%), who were not significantly different from controls [47]. Loading modality (high-impact, odd-impact, or repetitive non-impact) predicted 25.0% of the variance in distal tibial cortical wall thickness.

A Finnish birth cohort analysis revealed that leisure time physical activity between the ages of fourteen and 46 was associated with larger vertebral cross sectional area in women but not men [48]. The relationship was most evident at the highest dose, as no differences were observed between inactive and moderately active women, and only women who were physically active four or more times per week aged 31 exhibited significant differences from the inactive group at 46 years of age.

A number of research instruments have been developed to record physical activity participation of specific relevance to bone, that is, according to the intensity of engendered skeletal strain (low, moderate, or high). Weight-bearing load, frequency of loading, and duration of activity can be used to assign a peak strain score [49], an ‘Osteogenic Index’ [50], a ‘Bone-specific Physical Activity’ score [51], or ‘Bone Loading Unit’ [52]. For example, the ‘Osteogenic Index’ algorithm was used to categorise lifetime loading history of community-dwelling men over 50 into high (H) or low to non-impact (L) during two age periods: thirteen to eighteen years (adolescence) and nineteen to >50 years (adulthood) [53]. Participants were divided into three groups: LL, HL, and HH according to the ‘Osteogenic Index’ for each age period. QCT-derived total and cortical bone CSA at the femoral mid-shaft were significantly greater for men with a HH versus LL loading history (*p* < 0.05). By contrast, no relationship was observed between lifetime ‘Osteogenic Index’ and DXA-derived aBMD at the femoral neck or QCT-derived cortical vBMD at the mid-femur.

In summary, observational findings suggest weight-bearing physical activity may influence bone strength due to favourable geometric adaptation, independent of changes in aBMD.

## 4. Exercise Intervention Trials and Bone Geometry during Childhood and Adolescence

The influence of exercise interventions on growing bone has attracted a degree of interest in recent years owing to the theoretical assumption that maximising peak bone mass in youth will prevent fracture in older age. Paediatric exercise interventions have predominantly used standard two-dimensional DXA to determined aBMD and bone mineral content (BMC; grams) at the lumbar spine and/or proximal femur, and estimated bone geometric parameters such as CSA and cortical thickness at the hip using specialised hip strength analysis software [54]. As mentioned previously, bone size affects DXA-derived aBMD, thus normal changes in bone size over time in growing children confound DXA interpretation. For this reason, BMC has been more commonly reported than aBMD in longitudinal paediatric exercise intervention trials. While the use of pQCT, QCT, and MRI is rare in such trials, quantification of volumetric density, bone size, and bone geometry enhances our understanding of the effects of exercise on skeletal health. Although paediatric interventions have been heterogeneous in terms of exercise program frequency and duration, most have implemented school-based jumping activities based on the fundamental principles of osteogenic loading and ease of implementation. Of the studies outlined below in children and adolescents, only the ‘CAPO Kids’ trial [55,56] included changes in pQCT-derived geometry of both weight-bearing and non-weight-bearing bones, while the majority of studies reported lower extremity sites only [57,58,59,60].

### 4.1. Girls

The first controlled school-based exercise intervention to examine bone geometry involved 126 pre- to postmenarcheal girls aged ten to fifteen years performing nine months of twice-weekly non-impact aerobic exercise with multidirectional jumps, or control activities [57]. While changes in lumbar spine and femoral neck BMC in premenarcheal girls were significantly higher for the intervention group than control, (+8.6% vs. +5.3%, *p* = 0.012; +9.3% vs. +5.3%, *p* = 0.014, respectively), tibial mid-shaft cortical CSA was not significantly affected (+6.0% vs. +4.4%, respectively, *p* > 0.05). In this growing paediatric cohort, high-impact jumping resulted in exercise-induced effects at predominantly trabecular but not cortical bone sites, and the responses differed depending on growth velocity. The authors proposed that rapid expansion of the metaphyseal region of growing bones in premenarcheal girls may have reduced the capacity for adaptation at weight-bearing cortical sites. The lack of effect at cortical bone sites may be attributable to the relatively low box jump height of 30 cm, with jumps on and off the box likely imparting inadequately high magnitude cortical bone strain.

### 4.2. Combined Girls and Boys

The ‘Action Schools! BC’ 16-month cluster randomised trial implemented a bone-loading jumping program (‘Bounce at the Bell’), supplementary to standard physical education classes, in prepubertal and peripubertal children (10.2 ± 0.6 years) [58]. Prepubertal boys exposed to the intervention tended to increase tibial mid-shaft cortical bone CSA compared with maturity-matched control boys, but the effect was not significant (*p* > 0.05). The program was not effective for distal tibia or mid-shaft bone CSA in girls of any maturation status, nor for early pubertal boys. It was hypothesised that the lack of geometric adaptation may be attributable to the jumps being of insufficient intensity, frequency (15 to 36 jumps per day), or duration (three-mins per session) to act as an adequately novel stimulus in highly active children already performing six hours of weight-bearing physical activity per week. Localised adaptation in mid-shaft tibial cortical CSA and thickness across bone quadrants (anterior, posterior, medial, and lateral) for a subsample of boys aged nine to eleven years (139 intervention and 63 control) was reported separately [59]. Percent change in cortical CSA and thickness across quadrants was not significantly different between groups. Intervention boys tended to increase geometric parameters in the anterior and medial quadrants; however, again, the changes were not significant (*p* > 0.05).

A twelve-week high-impact jumping program in three- to eighteen-year-old children (intervention: seventeen girls, eleven boys; control: fourteen girls, twelve boys) increased pQCT-derived distal tibial BMC and vBMD (but not periosteal and endosteal circumference, or cortical and total CSA) in peripubertal children, but not other maturity stages [60]. The outcomes must be interpreted with caution, however, as program compliance was highly variable (ranging from 11.0% to 96.0%, mean 76.0%), and the twelve-week intervention period was unlikely to have been adequate to stimulate a measurable bone response [61].

The ‘CAPO Kids’ study implemented a ten-minute, thrice-weekly instructor-led capoeira (a Brazilian sport combining martial arts and dance) and jumping intervention, alongside standard physical education classes, in year five and six school children [55,56]. A subsample of boys and girls underwent pQCT. Positive changes were observed in calcaneal broadband ultrasound attenuation for both boys (intervention +4.3% vs. control +2.1%, *p* = 0.001) and girls (intervention +4.9% vs. control +1.4%, *p* = 0.019), however, only radial diaphyseal periosteal circumference increased to a greater extent in the intervention group than control (+9.2% vs. +4.8%, *p* < 0.05), and a trend for increased cortical CSA in the exercise group was observed (+12.4% vs. +7.7%, *p* > 0.05) in boys only. It is likely the subgroup sample size for both sexes was underpowered to detect an exercise effect on bone geometry from pQCT measures.

## 5. Exercise Intervention Trials and Bone Geometry in Young and Middle-Aged Adults

### 5.1. Premenopausal Women

To our knowledge, only one study examining the response of parameters of bone geometry to exercise intervention in young- to middle-aged adult women has been undertaken [62]. A twelve-month impact exercise regimen for 35- to 40-year-old healthy premenopausal women increased mid-femur bone circumference by 0.2% (*p* = 0.033) but not proximal tibial bone circumference, cortical thickness, or CSA. Results appear to have been influenced by compliance. Those in the highest quintile for attendance (>66 sessions) increased tibial circumference and cortical CSA by 1.2% and 0.5%, respectively, compared with the lowest quintile (<19 sessions). Furthermore, load intensities of >3.9 times gravity were related to change in mid-femur bone circumference, cortical CSA, and cortical thickness (*r* = 0.372–0.446, *p* < 0.05). As no baseline *T*-scores were reported, it is not clear if the participants had normal, osteopenic, or osteoporotic bone mass. The principle of initial values, whereby the skeletal response to an exercise stimulus is typically greatest in those with the lowest initial values, may partly account for the minimal exercise-induced gains observed, even in the most compliant women. To illustrate, following a 12-month training program of thrice-weekly resistance plus impact training, premenopausal women with the lowest initial aBMD exhibited the greatest magnitude increase at the proximal femur [63]. Overall, young healthy individuals with ‘average’ baseline bone mass are unlikely to display notable changes in parameters of bone strength.

### 5.2. Young Adult Men

We are aware of only two exercise studies reporting bone geometry in young adult men, both of which were conducted in military cohorts [64,65]. The ‘Lichfield’ study [65] examined the skeletal response to a twelve-week British Army basic training regimen in 399 healthy male recruits (19.9 ± 2.4 years). Training, including multiple 40 to 80-min sessions of progressive resistance training, circuit training, endurance training, agility training, and sports increased MRI-derived right and left femoral shaft periosteal (0.78 ± 3.14% and 0.59 ± 2.58%, *p* < 0.001, respectively) and cortical volume (1.09 ± 4.05% and 0.71 ± 4.05%, *p* < 0.05, respectively). A more recent ten-week military training program (including runs, loaded marches, swimming, and military specific drills) increased cortical CSA, total CSA, and cortical thickness at the tibial mid-shaft (*p* < 0.05) in 90 British Army recruits (21 ± 3 years) [64]. Findings support that high intensity military training can enhance bone geometry at the femoral and tibial mid-shaft in young healthy men, even in the short term.

There are several caveats to the aforementioned observations: (1) comparison/control groups were not present; (2) burdensome and intensive military-style training programs are unlikely to be appropriate for general public implementation; (3) young healthy male Army recruits are not entirely representative of the general population; and (4) high-volume military training is associated with an elevated risk of bone stress injury.

### 5.3. Combined Premenopausal Women and Young Adult Men

The only combined study of young adult men and women to examine the influence of exercise on bone geometry was a very small (*n* = 22), short duration (sixteen weeks, three sessions per week), three-arm comparison of either squats plus deadlifts (three men, four women), hip adduction plus hip abduction (three men, five women), or a combination of both (three men, four women) in previously sedentary men and women aged 22 to 55 years [66]. Hip adduction/abduction led to an increase in trochanteric cortical volume (+4.1%, *p* < 0.01), and squat and deadlift exercise increased femoral neck cortical volume (+1.6%, *p* < 0.05), but combined exercise had no effect (*p* > 0.05). The somewhat non-intuitive findings can likely be explained by inadequate study design quality, and therefore require replication.

## 6. Exercise Intervention Trials and Bone Geometry in Older Adults

While exercise interventions in paediatric cohorts have been primarily jump-based programs, exercise protocols in older adult trials have varied by mode, intensity, frequency, and duration. Thus, for the purpose of the current review, studies in older adults have been categorised according to sex and exercise mode (weight-bearing aerobic exercise, impact loading, resistance training, and combination training).

### 6.1. Older Men

Owing to the higher prevalence of osteoporosis in women, investigations into exercise interventions for bone mass and strength in postmenopausal women have predominated, with little work done in middle-aged and older men. Thus, the effects of exercise on bone geometry in older men remain to be fully determined.

#### 6.1.1. Weight-Bearing Aerobic Exercise

No studies have been conducted on the effects of weight-bearing aerobic exercise on bone geometry in middle-aged and older men. Controlled or randomised controlled trials of running [67,68] and brisk walking [69,70] in this population have utilised only single-photon absorptiometry at the calcaneus or DXA at the lumbar spine and proximal femur to determine aBMD and BMC. Results suggest weight-bearing aerobic exercise is not an effective stimulus to maintain or elevate aBMD at clinically relevant sites, but the effect on bone geometry is unknown.

#### 6.1.2. Impact Loading

The ‘Hip-Hop‘ study examined twelve months of 50 daily multidirectional hops, in 34 apparently healthy men, aged 65 to 80 years utilising a unique within-subjects unilateral loading design. The stimulus was sufficient to elicit gains in mid-femoral neck CSA in both the loaded (+2.0%) and unloaded (+2.1%) legs [71]. Proximal femur thickness tended to increase in the exercise leg and decrease in the control leg (+0.5% vs. −0.2%, *p* > 0.05), however, the net +0.8% difference between exercise and control legs was not significant (*p* = 0.24). While the within-subject unilateral design has notable strengths, it also has limitations, including not completely partitioning the effects of systemic agents stimulated by exercise that may influence the whole skeleton. Whether individuals had normal or low bone mass at baseline was not reported.

#### 6.1.3. Resistance Training

Although exercise guidelines for healthy adults or those with low bone mass recommend progressive resistance training (PRT) for all major muscle groups [33,34,72], no randomised, controlled trial of PRT alone has reported the response of bone geometric outcomes. PRT has, however, been examined in combination with weight-bearing exercise.

#### 6.1.4. Combined Aerobic, Resistance, and Impact Training

Eighteen months of combined PRT, aerobic, and impact training did not enhance QCT-derived indices of bone strength in men aged 50 to 79 years [73]. Adherence to the exercise program was moderate (63%) and the highly variable resistance training intensity across the course of the study may partially explain findings. As individuals with osteoporosis (*T*-score ≤ −2.5) were excluded, it is unknown if a more marked effect would have been observed in men with lower initial bone mass.

### 6.2. Postmenopausal Women

The value of exercise as a non-pharmacological strategy for osteoporosis prevention and rehabilitation has been a topic of much investigation over the last decades, due to the high prevalence of osteoporosis in postmenopausal women. Multiple systematic reviews and meta-analyses have synthesised evidence from exercise intervention trials to evaluate the effect on aBMD at the lumbar spine and/or proximal femur [27,28,29,30,31,32]. Overall, much of the evidence suggests resistance training, weight-bearing impact exercise, or a combination of the two will prevent postmenopausal bone loss at clinically relevant sites. Studies examining morphological changes are limited, however, and few systematic reviews have been undertaken [74,75,76].

#### 6.2.1. Weight-Bearing Aerobic Exercise

To date, there have been no studies examining the response of bone geometry to weight-bearing aerobic exercise alone in postmenopausal women. In keeping with the basic rules of bone adaptation that mechanical strains above those habitually experienced are required, it is unsurprising that walking has been found to be essentially ineffective for enhancing aBMD [77].

#### 6.2.2. Impact Loading

A twelve-month randomised placebo-controlled trial of antiresorptive drug therapy and multidirectional jumping and bench stepping on bone structure in 164 postmenopausal women observed an increase in lumbar spine (+1.3%) and femoral neck (+3.5%) aBMD with drug therapy alone, compared with control (*p* < 0.05) [78]. Exercise did not enhance the effect on bone mass, but increased the ratio of cortical bone to total bone area at the distal tibia by 3.7% compared with the non-exercise group. The enrolment of women one to five years post-menopause (i.e., a period of elevated bone loss) and moderate training session attendance (1.6 ± 0.9 per week) may have dampened the exercise effect.

#### 6.2.3. Resistance Training

No trials of resistance training alone have reported bone geometry outcomes in postmenopausal women. Most training protocols have combined resistance training with balance or impact-type activities. A meta-analysis examining the effects of progressive, “high-intensity” resistance training interventions (eight to twelve repetitions, corresponding to 60% to 70% of one repetition maximum) on changes in aBMD in postmenopausal women reported a significant training effect at the lumbar spine, a non-significant positive effect at the total hip, but no effect at the femoral neck [32].

#### 6.2.4. Combined Aerobic, Resistance, and Impact Training

A six-month twice-weekly program of supervised resistance training and impact loading, with additional home training of similar exercise to the supervised program, designed specifically to load the wrist, provoked no effect from the proximal radius cortical CSA, the distal radius total CSA, or the distal radius trabecular CSA in postmenopausal women aged 52 to 72 years [79]. By contrast, the distal radius cortical CSA of exercisers increased by 2.8% compared with a 0.2% loss for controls (*p* = 0.05), with expansion of cortical area indicative of periosteal apposition in response to mechanical loading. It is not clear whether participants were osteoporotic or normal bone status.

A twelve-month trial of supervised circuit training (lower body impact and upper body resistance training) with additional home training (lower body impact, and core strengthening exercises), hormone replacement therapy (HRT), or a combination of the two, found circuit training increased bone mass at the posterior region of the proximal tibia, while HRT and HRT plus circuit training improved the anteroposterior region in early postmenopausal women [80]. The most pronounced site-specific changes in polar bone mass distribution at the femoral and tibial shafts occurred for HRT plus exercise (*p* < 0.05). The moderate exercise effect may be attributable to lower session attendance in the exercise-alone group (average 1.0 per week) compared with the HRT plus exercise group (approximately 1.2 to 1.3 sessions per week).

A 25-week twice-weekly trial of PRT (machine and free weights), agility training (ball games, relays, dance, and obstacle courses), or ‘sham’ exercise (stretching, deep breathing, and relaxation) observed that vBMD at the tibial shaft improved in response to agility training targeting the lower extremities, and at the radial shaft in response to upper extremities resistance training (bicep curls and tricep extensions), but no changes in tibial or radial cortical or total CSA occurred for any exercise group, in 75- to 85-year-old community-dwelling women [81]. This study included women with both osteoporosis and osteopenia at the lumbar spine or total hip (*T*-score < −1.0), on or off bisphosphonates.

Previously sedentary women aged 70 to 79 years were randomly assigned to twelve-month supervised PRT, balance-jumping training, combined PRT and balance-jump training, or a control group [82]. Per protocol analyses (more than two sessions per week) revealed a trend for pQCT-derived tibial mid-shaft cortical CSA to increase for combined training only (*p* = 0.062). The lack of exercise effect seen in the intention to treat analyses could be partly attributable to the moderate training compliance for resistance (74%), balance-jumping (59%), and combined training programs (67%). A subsequent paper reported there were no residual bone geometric benefits one year after cessation of the exercise intervention in a subset of the original study participants [83]. Women with a femoral neck BMD *T*-score < −2.5 were excluded, limiting the applicability of findings to osteoporotic postmenopausal women.

## 7. Summary

Substantial cross-sectional observations suggest that physical activity influences bone mass and strength at all ages of skeletal development. Those data would suggest that regular physical activity likely promotes bone mass accrual and optimisation of bone geometry during childhood, consolidates or aids in the maintenance of bone during adulthood, and maintains or attenuates the loss of bone mass and strength during old age, thereby theoretically reducing the risk of osteoporotic fracture at the end of life. Randomised controlled intervention trials confirm that bone-targeted exercise programs can positively influence aBMD and BMC of loaded bones. As few high quality randomised controlled trials have examined the response of bone geometry to such exercise programs, however, questions remain in that area. Adaptations in bone geometry are most evident after interventions that apply either a very novel (e.g., to the upper extremity [55,56,79]) or very intense (e.g., military training [64,65]) form of loading. The response is most commonly an increase in periosteal diameter, cortical thickness, and/or cross sectional area. Nevertheless, our interpretation of the literature aligns with the recent scientific statement from the National Osteoporosis Foundation that while there is currently strong (Level A) evidence to support the positive effect of physical activity on bone mass, evidence supporting the effect of physical activity on bone structure is less compelling (Level B) [84].

The dearth of appropriately designed randomised controlled exercise intervention trials to have examined the effect of exercise training on bone geometry using three-dimensional scanning devices is highlighted by the limited number of studies available to include in the current review. Of the latter, four were conducted in children and adolescents, and four in premenopausal women and young adult men. In older adults, the population most likely to benefit from exercise as a non-pharmacological strategy for the management of osteoporosis, only two trials have been undertaken in older men and five in older women. Direct comparisons between trials is complicated by considerable study heterogeneity (age, sex, and health status of participants, exercise program mode, frequency, intensity, duration, and intervention length, scanning methodology, skeletal sites measured), as well as a multitude of evident design flaws (inappropriate inclusion/exclusion criteria, randomisation procedures, and statistical analyses). It is also not clear whether existing measurement techniques (such as pQCT) are sufficiently sensitive to detect the potentially very subtle changes in bone geometry that may impart notable strength benefits to whole bones. While preliminary evidence is promising, high quality trials are urgently required.

## 8. Future Research

There are a number of targets for future research attention, and many important considerations for the design of future studies to avoid repeating past methodological mistakes. As more data is needed for every age and sex, it is clear that future exercise interventions in all populations should include measures of bone geometry at clinically-relevant sites as standard practice. Although lifetime risk of osteoporotic fracture is lower in men, they are more likely to suffer serious post-fracture consequences, thus the need for more data on men is particularly apparent. Individuals most at risk of low trauma fracture, such as those with osteopenia and osteoporosis and a history of fragility fracture, should also be targeted. The interaction of exercise with anti-osteoporosis medication requires further attention. That women with osteoporosis or previous fracture, and those taking anti-osteoporosis medications have frequently been excluded from randomised, controlled trials limits the generalisability of findings to the demographic of most relevance to the findings. There is also a need for trials of sufficient length (more than six months) to ensure a change in bone geometry can be detected with existing imaging techniques. Concerning exercise mode, not all types of exercise provide an effective stimulus for bone, with walking and non-weight-bearing activities being particularly ineffective. To initiate an osteogenic response, mechanical loading must induce bone strains that are considerably greater than habitually experienced. Exercise interventions that do not achieve such loading are unlikely to be effective nor contribute to the body of knowledge in the field. Studies that allow the determination of exercise dose response will facilitate the development of appropriate exercise recommendations for bone health. It is clear that future exercise research should monitor changes in bone geometry beyond DXA-derived aBMD to provide further insight into the effects of mechanical loading on bone morphology, and thus bone strength, and risk of osteoporotic fracture.

## References

[1] Bouillon R., Burckhardt P., Christiansen C., Fleisch H.A., Fujita T., Gennari C., Marin T.J., Mazzuoli G., Melton L.J., Ringe J.D. (1991). Consensus development conference: Prophylaxis and treatment of osteoporosis. Am. J. Med..

[2] Reginster J.Y., Burlet N. (2006). Osteoporosis: A still increasing prevalence. Bone.

[3] Wright N.C., Looker A.C., Saag K.G., Curtis J.R., Delzell E.S., Randall S., Dawson-Hughes B. (2014). The recent prevalence of osteoporosis and low bone mass in the United States based on bone mineral density at the femoral neck or lumbar spine. J. Bone. Miner Res..

[4] Henry M.J., Pasco J.A., Nicholson G.C., Kotowicz M.A. (2011). Prevalence of osteoporosis in Australian men and women: Geelong Osteoporosis Study. Med. J. Aust..

[5] Svedbom A., Ivergard M., Hernlund E., Rizzoli R., Kanis J.A. (2014). Epidemiology and economic burden of osteoporosis in Switzerland. Arch. Osteoporos..

[6] Hernlund E., Svedbom A., Ivergard M., Compston J., Cooper C., Stenmark J., McCloskey E.V., Jonsson B., Kanis J.A. (2013). Osteoporosis in the European Union: Medical management, epidemiology and economic burden. Arch. Osteoporos..

[7] Kanis J.A. (1994). Assessment of fracture risk and its application to screening for postmenopausal osteoporosis: Synopsis of a WHO report. Osteoporos. Int..

[8] Johnell O., Kanis J.A. (2006). An estimate of the worldwide prevalence and disability associated with osteoporotic fractures. Osteoporos. Int..

[9] Oden A., McCloskey E.V., Kanis J.A., Harvey N.C., Johansson H. (2015). Burden of high fracture probability worldwide: Secular increases 2010–2040. Osteoporos. Int..

[10] Cummings S.R., Melton L.J. (2002). Epidemiology and outcomes of osteoporotic fractures. Lancet.

[11] Johnell O., Kanis J. (2005). Epidemiology of osteoporotic fractures. Osteoporos. Int..

[12] Melton L.J., Chriscilles E.A., Cooper C., Lane W.A., Riggs B.L. (1992). Perspective: How many women have osteoporosis?. J. Bone Miner. Res..

[13] Kanis J.A., Johnell O., Oden A., Sernbo I., Redlund-Johnell I., Dawson A., De Laet C., Jonsson B. (2000). Long-term risk of osteoporotic fracture in Malmö. Osteoporos. Int..

[14] Hiligsmann M., Bruyère O., Ethgen O., Gathon H.J., Reginster J.Y. (2008). Lifetime absolute risk of hip and other osteoporotic fracture in Belgian women. Bone.

[15] Doherty D.A., Sanders K.M., Kotowicz M.A., Prince R.L. (2001). Lifetime and five-year age-specific risk of first and subsequent osteoporotic fractures in postmenopausal women. Osteoporos. Int..

[16] Mosley J.R., March B.M., Lynch J., Lanyon L.E. (1997). Strain magnitude related changes in whole bone architecture in growing rats. Bone.

[17] Hsieh Y.-F., Robling A.G., Ambrosius W.T., Burr D.B., Turner C.H. (2001). Mechanical loading of diaphyseal bone in vivo: The strain threshold for an osteogenic response varies with location. J. Bone Miner. Res..

[18] O’Connor J.A., Lanyon L.E., MacFie H. (1982). The influence of strain rate on adaptive bone remodelling. J. Biomech..

[19] Rubin C.T., McLeod K.J. (1994). Promotion of bony ingrowth by frequency-specific, low-amplitude mechanical strain. Clin. Orthop. Relat. Res..

[20] Hsieh Y.F., Turner C.H. (2001). Effects of loading frequency on mechanically induced bone formation. J. Bone Miner. Res..

[21] Rubin C.T., Turner A.S., Mallinckrodt C., Jerome C., McLeod K.J., Bain S. (2002). Mechanical strain, induced noninvasively in the high-frequency domain, is anabolic to cancellous bone, but not cortical bone. Bone.

[22] Rubin C.T., Lanyon L.E. (1984). Regulation of bone formation by applied dynamic loads. J. Bone Joint Surg. Am..

[23] Lanyon L.E., Rubin C.T. (1984). Static vs. dynamic loads as an influence on bone remodelling. J. Biomech..

[24] Kelley G.A., Kelley K.S., Khort W.M. (2013). Exercise and bone mineral density in men: A meta-analysis of randomized controlled trials. Bone.

[25] Bolam K.A., Van Uffelen J.G.Z., Taaffe D.R. (2013). The effect of physical exercise on bone density in middle-aged and older men: A systematic review. Osteoporos. Int..

[26] Kelley G.A., Kelley K.S., Tran Z.V. (2000). Exercise and bone mineral density in men: A meta-analysis. J. Appl. Physiol..

[27] Howe T.E., Shea B., Dawson L.J., Downie F., Murray A., Ross C., Harbour R.T., Caldwell L.M., Creed G. (2011). Exercise for preventing and treating osteoporosis in postmenopausal women. Cochrane Database Syst. Rev..

[28] Zhao R., Zhao M., Xu Z. (2015). The effects of differing resistance training modes on the preservation of bone mineral density in postmenopausal women: A meta-analysis. Osteoporos. Int..

[29] Martyn-St James M., Carroll C. (2008). A meta-analysis of impact exercise on postmenopausal bone loss: The case for mixed loading exercise programmes. Br. J. Sports Med..

[30] Wallace B.A., Cumming R.G. (2000). Systematic review of randomized trials of the effect of exercise on bone mass in pre- and postmenopausal women. Calcif. Tissue Int..

[31] Zehnacker C.H., Bemis-Dougherty A. (2007). Effect of weighted exercises on bone mineral density in post menopausal women. A systematic review. J. Geriatr. Phys. Ther..

[32] Martyn-St James M., Carroll S. (2006). High-intensity resistance training and postmenopausal bone loss: A meta-analysis. Osteoporos. Int..

[33] Giangregorio L.M., Papaioannou A., Macintyre N.J., Ashe M.C., Heinonen A., Shipp K., Wark J.D., McGill S., Keller H., Jain R. (2014). Too Fit To Fracture: Exercise recommendations for individuals with osteoporosis or osteoporotic vertebral fracture. Osteoporos. Int..

[34] Beck B.R., Daly R.M., Singh M.A., Taaffe D.R. (2016). Exercise and Sports Science Australia (ESSA) position statement on exercise prescription for the prevention and management of osteoporosis. J. Sci. Med. Sport.

[35] Edwards M.H., Jameson K., Denison H., Harvey N.C., Sayer A.A., Dennison E.M., Cooper C. (2013). Clinical risk factors, bone density and fall history in the prediction of incident fracture among men and women. Bone.

[36] Pasco J.A., Seeman E., Henry M.J., Merriman E.N., Nicholson G.C., Kotowicz M.A. (2006). The population burden of fractures originates in women with osteopenia, not osteoporosis. Osteoporos. Int..

[37] Kanis J.A. (2002). Diagnosis of osteoporosis and assessment of fracture risk. Lancet.

[38] Guglielmi G., Muscarella S., Bazzocchi A. (2011). Integrated imaging approach to osteoporosis: State-of-the-art review and update. Radiographics.

[39] Bono C.M., Einhorn T.A. (2003). Overview of osteoporosis: Pathophysiology and determinants of bone strength. Eur. Spine J..

[40] Tan V.P.S., Macdonald H.M., Kim S., Nettlefold L., Gabel L., Ashe M.C., McKay H.A. (2014). Influence of physical activity on bone strength in children and adolescents: A systematic review and narrative synthesis. J. Bone Miner. Res..

[41] Ducher G., Daly R.M., Bass S.L. (2009). Effects of repetitive loading on bone mass and geometry in young male tennis players: A quantitative study using MRI. J. Bone Miner. Res..

[42] Bass S.L., Saxon L., Daly R.M., Turner C.H., Robling A.G., Seeman E., Stuckey S. (2002). The effect of mechanical loading on the size and shape of bone in pre-, peri-, and postpubertal girls: A study in tennis players. J. Bone Miner. Res..

[43] Weatherholt A.M., Warden S.J. (2016). Tibial bone strength is enhanced in the jump leg of collegiate-level jumping athletes: A within-subject controlled cross-sectional study. Calcif. Tissue Int..

[44] Bradshaw E.J., Le Rossignol P. (2004). Anthropometric and biomechanical field measures of floor and vault ability in 8 to 14 year old talent-selected gymnasts. Sports Biomech..

[45] Dowthwaite J.N., Scerpella T.A. (2011). Distal radius geometry and skeletal strength indices after peripubertal artistic gymnastics. Osteoporos. Int..

[46] Rantalainen T., Duckham R.L., Suominen H., Heinonen A., Alen M., Korhonen M.T. (2014). Tibial and fibular mid-shaft bone traits in young and older sprinters and non-athletic men. Calcif. Tissue Int..

[47] Nikander R., Sievänen H., Uusi-Rasi K., Heinonen A., Kannus P. (2006). Loading modalities and bone structures at nonweight-bearing upper extremity and weight-bearing lower extremity: A pQCT study of adult female athletes. Bone.

[48] Oura P., Paananen M., Niinimaki J., Tammelin T., Herrala S., Auvinen J., Korpelainen R., Junno J.A., Karppinen J. (2016). Effects of leisure-time physical activity on vertebral dimensions in the Northern Finland Birth Cohort 1966. Sci. Rep..

[49] Kemper H.C., Bakker I., Twisk J.W., van Mechelen W. (2002). Validation of a physical activity questionnaire to measure the effect of mechanical strain on bone mass. Bone.

[50] Turner C.H., Robling A.G. (2003). Designing exercise regimens to increase bone strength. Exerc. Sport Sci. Rev..

[51] Weeks B.K., Beck B.R. (2008). The BPAQ: A bone-specific physical activity assessment instrument. Osteoporos. Int..

[52] Dolan S.H., Williams D.P., Ainsworth B.E., Shaw J.M. (2006). Development and reproducibility of the bone loading history questionnaire. Med. Sci. Sports Exerc..

[53] Daly R.M., Bass S.L. (2006). Lifetime sport and leisure activity participation is associated with greater bone size, quality and strength in older men. Osteoporos. Int..

[54] Beck T.J., Ruff C.B., Warden S.J., Scott W.W., Rao G.U. (1990). Predicting femoral neck strength from bone mineral data: A structural approach. Invest Radiol.

[55] Nogueira R.C., Weeks B.K., Beck B.R. (2014). An in-school exercise intervention to enhance bone and reduce fat in girls: The CAPO Kids trial. Bone.

[56] Nogueira R.C., Weeks B.K., Beck B.R. (2015). Targeting bone and fat with novel exercise for peripubertal boys: The CAPO kids trial. Pediatr. Exerc. Sci..

[57] Heinonen A., Sievänen H., Kannus P., Oja P., Pasanen M., Vuori I. (2000). High-impact exercise and bones of growing girls: A 9-month controlled trial. Osteoporos. Int..

[58] Macdonald H.M., Kontulainen S.A., Khan K.M., McKay H.A. (2007). Is a school-based physical activity intervention effective for increasing tibial bone strength in boys and girls?. J. Bone Miner. Res..

[59] Macdonald H.M., Cooper D.M., McKay H.A. (2009). Anterior-posterior bending strength at the tibial shaft increases with physical activity in boys: Evidence for non-uniform geometric adaptation. Osteoporos. Int..

[60] Johannsen N., Binkley T., Englert V., Neiderauer G., Specker B. (2003). Bone response to jumping is site-specific in children: A randomized trial. Bone.

[61] Clarke B. (2008). Normal bone anatomy and physiology. Clin. J. Am. Soc. Nephrol..

[62] Vainionpää A., Korpelainen R., Sievänen H., Vihriälä E., Leppäluoto J., Jämsä T. (2007). Effect of impact exercise and its intensity on bone geometry at weight-bearing tibia and femur. Bone.

[63] Winters-Stone K., Snow C.M. (2003). Initial values predict musculoskeletal reponse to exercise in premenopausal women. Med. Sci. Sports Exerc..

[64] Izard R.M., Fraser W.D., Negus C., Sale C., Greeves J.P. (2016). Increased density and periosteal expansion of the tibia in young adult men following short-term arduous training. Bone.

[65] Eleftheriou K.I., Rawal J.S., Kehoe A., James L.E., Payne J.R., Skipworth J.R., Puthucheary Z.A., Drenos F., Pennell D.J., Loosemore M. (2012). The Lichfield bone study: The skeletal response to exercise in healthy young men. J. Appl. Physiol..

[66] Lang T.F., Saeed I.H., Streeper T., Carballido-Gamio J., Harnish R.J., Frassetto L.A., Lee S.M., Sibonga J.D., Keyak J.H., Spiering B.A. (2014). Spatial heterogeneity in the response of the proximal femur to two lower-body resistance exercise regimens. J. Bone Miner. Res..

[67] Williams J.A., Wagner J., Wasnich R., Heilbrun L. (1984). The effect of long-distance running upon appendicular bone mineral content. Med. Sci. Sports Exerc..

[68] Michel B.A., Lane N.E., Björkengren A., Bloch D.A., Fries J.F. (1992). Impact of running on lumbar bone density: A 5-year longitudinal study. J. Rheumatol..

[69] Huuskonen J., Väisänen S.B., Kröger H., Jurvelin J.S., Alhava E., Rauramaa R. (2001). Regular physical exercise and bone mineral density: A four-year controlled randomized trial in middle-aged men. The DNASCO study. Osteoporos. Int..

[70] Paillard T., Lafont C., Costes-Salon M.C., Riviere D., Dupui P. (2004). Effects of brisk walking on static and dynamic balance, locomotion, body composition, and aerobic capacity in ageing healthy active men. Int. J. Sports Med..

[71] Allison S.J., Poole K.E., Treece G.M., Gee A.H., Tonkin C., Rennie W.J., Brooke-Wavell K. (2015). The influence of high impact exercise on cortical and trabecular bone mineral content and 3D distribution across the proximal femur in older men: A randomised controlled unilateral intervention. J. Bone Miner. Res..

[72] Garber C.E., Blissmer B., Deschenes M.R., Franklin B.A., Lamonte M.J., Lee I.M., Nieman D.C., Swain D.P. (2011). ‘American College of Sports Medicine’ position stand. Quantity and quality of exercise for developing and maintaining cardiorespiratory, musculoskeletal, and neuromotor fitness in apparently healthy adults: Guidance for prescribing exercise. Med. Sci. Sports Exerc..

[73] Kukuljan S., Nowson C.A., Sanders K.M., Nicholson G.C., Seibel M.J., Salmon J., Daly R.M. (2011). Independent and combined effects of calcium-vitamin D3 and exercise on bone structure and strength in older men: An 18-month factorial design randomized controlled trial. J. Clin. Endocrinol. Metab..

[74] Nikander R., Sievanen H., Heinonen A., Daly R.M., Uusi-Rasi K., Kannus P. (2010). Targeted exercise against osteoporosis: A systematic review and meta-analysis for optimising bone strength throughout life. BMC Med..

[75] Hamilton C.J., Swan V.J., Jamal S.A. (2010). The effects of exercise and physical activity participation on bone mass and geometry in postmenopausal women: A systematic review of pQCT studies. Osteoporos. Int..

[76] Polidoulis I., Beyene J., Cheung A.M. (2012). The effect of exercise on pQCT parameters of bone structure and strength in postmenopausal women: A systematic review and meta-analysis of randomized controlled trials. Osteoporos. Int..

[77] Palombaro K.M. (2005). Effects of walking-only interventions on bone mineral density at various skeletal sites: A meta-analysis. J. Geriatr. Phys. Ther..

[78] Uusi-Rasi K., Kannus P., Cheng S., Sievanen H., Pasanen M., Heinonen A., Nenonen A., Halleen J., Fuerst T., Genant H. (2003). Effect of alendronate and exercise on bone and physical performance of postmenopausal women: A randomized controlled trial. Bone.

[79] Adami S., Gatti D., Braga V., Bianchini D., Rossini M. (1999). Site-specific effects of strength training on bone structure and geometry of ultradistal radius in postmenopausal women. J. Bone. Miner. Res..

[80] Cheng S., Sipilä S., Taaffe D.R., Puolakka J., Suominen H. (2002). Change in bone mass distribution induced by hormone replacement therapy and high-impact physical exercise in post-menopausal women. Bone.

[81] Liu-Ambrose T.Y., Khan K.M., Eng J.J., Heinonen A., McKay H.A. (2004). Both resistance and agility training increase cortical bone density in 75- to 85-year-old women with low bone mass: A 6-month randomized controlled trial. J. Clin. Densitom..

[82] Karinkanta S., Heinonen A., Sievanen H., Uusi-Rasi K., Pasanen M., Ojala K., Fogelholm M., Kannus P. (2007). A multi-component exercise regimen to prevent functional decline and bone fragility in home-dwelling elderly women: Randomized, controlled trial. Osteoporos. Int..

[83] Karinkanta S., Heinonen A., Sievanen H., Uusi-Rasi K., Fogelholm M., Kannus P. (2009). Maintenance of exercise-induced benefits in physical functioning and bone among elderly women. Osteoporos. Int..

[84] Weaver C.M., Gordon C.M., Janz K.F., Kalkwarf H.J., Lappe J.M., Lewis R., O’Karma M., Wallace T.C., Zemel B.S. (2016). The National Osteoporosis Foundation’s position statement on peak bone mass development and lifestyle factors: A systematic review and implementation recommendations. Osteoporos. Int..

